# 

**Published:** 1913-10-18

**Authors:** 


### CONTENTS.

| | PAGE |
|---|---|
| **German and English Medical Education** ... | 55 |
| **The Non-Panel Movement** ... ... ... ... ... | 56 |
| **Hospital and Institutional News,** including— ... | 57 |
| The Secretaryship of the David Lewis Hospital, Liverpool—Changes at the General Hospital, Wolverhampton—An American Hospital Ward in London—Diversion of an Ancient Charity—The Coliseum Charity Performance—Nemesis of the Small Isolation Hospital. | |
| **Hospital Statistics** ... ... ... ... ... | 63 |
| Some Criticisms and Suggestions. | |
| **The Value of Petrol at Operations** ... ... | 64 |
| **Recent Advances in Orthopædics** ... ... ... | 65 |
| **Administrative Difficulties in Small Isolation Hospitals** ... ... ... ... ... ... | 66 |
| By H. FRANKLIN PARSONS, M.D., D.P.H. | |
| **The Future Tuberculosis Institution Resident** | 69 |
| **Hospitals from the Patients' Point of View** ... | 70 |
| Some Appreciations and a Suggestion. | |
| **Relaxations and Hobbies** ... ... ... ... | 71 |
| **The Gas Exhibition** ... ... ... ... ... | 72 |
| Institutional Applications Demonstrated. | |
| **Telephones for Hospital Use** ... ... .. | 73 |
| **Gifts and Bequests** ... ... ... ... ... | 74 |
| **The Editor's Letter-Box** ... ... ... ... | 75 |
| Quinine as a Hypnotic—National Insurance: The Distribution of the Medical Benefit Fund—Mr. Percy Raiment and Mr. Gordon R. Ward. | |
| **Non-Panel and Panel Practice under the Insurance Act** ... ... ... ... ... | 76 |
| Present Tendencies and Future Developments. | |
| **The Institutional Worker** ... ... (Suppl.) | 1 |
| **Our Bureau of Information** ... ... ,, | 2 |
| **Obituary** ... ... ... ... ... ,, | 3 |
| **The Book Market** ... ... ... ,, | 3 |
| **Buildings Contemplated** ... ... ,, | 3 |

